# Ocular findings in pediatric candidates for cochlear implantation with bilateral sensorineural hearing loss: a brief report from northwestern Iran

**DOI:** 10.1186/s12886-026-05205-w

**Published:** 2026-08-10

**Authors:** Navid Sobhi, Hadi Vahedi, Hamidreza Ashayeri, Fatemeh Razmjooei, Ali Jafarizadeh, Ali Nouri, Aida Ariafar, Yalda Jabbari Moghaddam, Masoud Naderpour

**Affiliations:** 1 Translational Ophthalmology Research Center, Farabi Eye Hospital, Tehran University of Medical Sciences, Tehran, Iran; 2 Nikookari Eye Center, Tabriz University of Medical Sciences, Tabriz, Iran; 3 Research Center for Evidence-Based Medicine, Iranian EBM Centre: A JBI Centre of Excellence, Faculty of Medicine, Tabriz University of Medical Sciences, Tabriz, Iran; 4 Poostchi Ophthalmology Research Center, Department of Ophthalmology, Medical School, Shiraz University of Medical Sciences, Shiraz, Iran; 5 Faculty of Medicine, Tabriz University of Medical Sciences, Tabriz, Iran; 6 Department of Otolaryngology, Head and Neck Surgery, Tabriz University of Medical Sciences, Tabriz, Iran

**Keywords:** Sensorineural hearing loss, Cochlear implantation, Ocular abnormalities, Refractive errors, Strabismus, Pediatric ophthalmology, Visual screening, Bilateral SNHL, Fundus abnormalities, Multidisciplinary care

## Abstract

**Background:**

The presence of ocular abnormalities in patients with sensorineural hearing loss (SNHL) may affect children’s developmental milestones due to impairment of primary sensory inputs. This study aimed to evaluate the prevalence of different ophthalmologic abnormalities in pediatric patients with bilateral SNHL who were candidates for cochlear implantation (CI).

**Methods:**

We performed a retrospective medical chart review of 261 pediatric patients under 13 years of age with bilateral SNHL who were candidates for CI and were evaluated at a tertiary cochlear implantation referral center in northwestern Iran between March 21, 2017, and June 20, 2025. Ophthalmologic findings were assessed as the main outcome measures.

**Results:**

The overall prevalence of ocular findings was 19.5%. Cardiac abnormalities were observed in 17.6% of patients, and refractive errors were identified in 8.4%. Jaundice was the most common prenatal complication, and 36.8% of patients had a history of neurologic complications. Ocular, cardiologic, and neurologic manifestations occurred independently of one another, with no significant pairwise co-occurrence among the three systems.

**Conclusions:**

Our findings highlight the need for a multidisciplinary approach in candidates for CI with bilateral SNHL. Comprehensive ophthalmologic evaluation is essential in this population, with particular attention to refractive errors, ocular alignment, and fundus assessment. Because ocular, cardiologic, and neurologic findings occurred independently in the exploratory analysis, screening for each system should be pursued comprehensively and in parallel rather than selectively guided by findings in another system.

**Trial registration:**

Not applicable.

## Introduction

Hearing loss (HL) is estimated to affect 1% of children in low to middle-income countries [1]. Sensorineural hearing loss (SNHL) is defined as a HL due to cochlear and auditory nerve damage [2], occurs in 1:2000 neonates and 6:1000 children ≤ 18 years old [3]. Pediatric patients with SNHL may also suffer from coexisting ocular manifestations, which impact their developmental status. Some genetic syndromes, such as Usher syndrome or Norrie disease, are known causes of deaf-blindness [4]. Visual input supports speech and language development in children with cochlear implants (CI), with visual cues enhancing spoken word recognition alongside auditory input [5, 6]. Children with dual sensory impairment show heterogeneous language outcomes, showing that visual function should not be assumed intact during candidacy evaluation [7]. Timely detection of ocular abnormalities is therefore clinically important, as uncorrected visual deficits may compound the effects of hearing loss, increasing the risk of developmental delay and reducing quality of life [8]. Accordingly, this study aims to determine the prevalence of various ocular findings in patients with SNHL who are candidates for CI, alongside associated cardiologic and neurologic manifestations, and to evaluate the co-occurrence between these findings.

## Methods

This descriptive, prevalence-based, and cross-sectional study used the data of 261 pediatric patients with bilateral SNHL who underwent CI in the Cochlear Implantation Center of Tabriz, East Azerbaijan, Iran, between March 21, 2017, and June 20, 2025. We included all patients with SNHL and age < 13 whose caregivers gave written consent to participate and publish. Patients were excluded if the medical chart did not contain a documented ophthalmologic assessment; if the hearing loss was conductive or mixed rather than sensorineural or if the record was a duplicate of a previously included patient. The age of participants, age at diagnosis, gestational age, sex, patients’ past medical history, type of delivery, maternal history during pregnancy (e.g., obstetric complications), and parental consanguinity were collected to characterize the participants. Clinical examinations, collected from medical records, yielded the following data: results of auditory brainstem response (ABR), neurologic findings, cardiac findings, speech disorders, and ocular findings, including refractive errors and the presence of deaf-blindness (e.g., Usher syndrome). HL severity was categorized from ABR-estimated thresholds as severe (80–89 dB HL), severe-to-profound (90–94 dB HL), and profound (≥ 95 dB HL or no identifiable Wave V at the maximum stimulus level) [9, 10]. 

In an exploratory, post hoc analysis, we evaluated whether ocular findings co-occurred with cardiologic and neurologic manifestations among pediatric candidates for CI with bilateral SNHL. For this purpose, three binary indicators were defined for each participant, then, to explore whether systemic involvement was associated with ocular findings, we fitted a binary logistic regression model with presence of ocular findings as the dependent variable and neurologic (N) and cardiologic (C) indicators as independent variables, reporting odds ratios with 95% confidence intervals. Statistical significance was set at *p* < 0.05.

## Results

We analyzed 261 cochlear implant candidates, with a male-to-female ratio of 49.4% to 50.6% and a mean age of 33.49 ± 53.39 months. Most were born at term and by cesarean section. The most common maternal disease was thyroid disease (4.2%). 72.4% had a history of admission to the neonatal intensive care unit. Parental consanguinity was common (first-degree 41.0%, second-degree 16.9%), and a positive family history of deafness was reported in first- and second-degree relatives in 7.7% and 9.6%, respectively. Table 1 represents the characteristics of the included subjects. The mean age of diagnosis for SNHL was 16.64 ± 22.89 months. Based on the ABR results of 522 ears included in this study, 487 showed severe-to-profound HL with a left-to-right ratio (L/R) of 247/240, 28 had severe HL with a L/R of 11/17, and 7 suffered from profound HL with a L/R ratio of 3/4.

**Table 1:** Characteristics of included patients

Characteristic	No. (%)
**Sex**	
Male	129 (49.4%)
Female	132 (50.6%)
**Age**,** Mean (SD)**,** Months**	33.49 ± 53.39
**Age at diagnosis**,** Mean (SD)**,** Months**	16.64 ± 22.89
**NICU admission**	189 (72.4%)
**Delivery type**	
Normal vaginal delivery	113 (43.2%)
Cesarean section	148 (56.8%)
**Gestational age**	
Term	220 (84.2%)
Preterm	41 (15.8%)
**Parental consanguinity**	
None	110 (42.1%)
First	107 (41%)
Second	44 (16.9%)
**Number of other children with SNHL**	
One child	8 (3.1%)
Two children	2 (0.8%)
**Family history of deafness**	
First degree	20 (7.7%)
Second degree	25 (9.6%)
**Maternal medical history**	
Thyroid disorders	11 (4.2%)
Infections	6 (2.3%)
Hypertensive disorders	5 (1.9%)
Diabetes	5 (1.9%)
Gynecologic (pregnancy-related)	4 (1.6%)
Hematologic /Gastroenterology	4 (1.6%)
Autoimmune /Rheumatologic	2 (0.8%)
Neurologic	2 (0.8%)
Renal (non-infectious)	1 (0.4%)

The most common complications observed in patients were neurologic (*n* = 96, 36.8%), followed by perinatal (*n* = 77, 29.5%), ophthalmologic (*n* = 51, 19.5%), and cardiovascular (*n* = 46, 17.6%). Other observed complications include infection (*n* = 18, 6.9%), renal or urological (*n* = 9, 3.4%), gastrointestinal (*n* = 6, 2.3%), endocrine (*n* = 6, 2.3%), and immunologic (*n* = 5, 1.9%). Usher syndrome was the most frequent genetic syndrome (*n* = 4, 1.5%). One patient was diagnosed with Bartter syndrome (*n* = 1, 0.4%) and one with Waardenburg syndrome (*n* = 1, 0.4%).

**Fig. 1:**
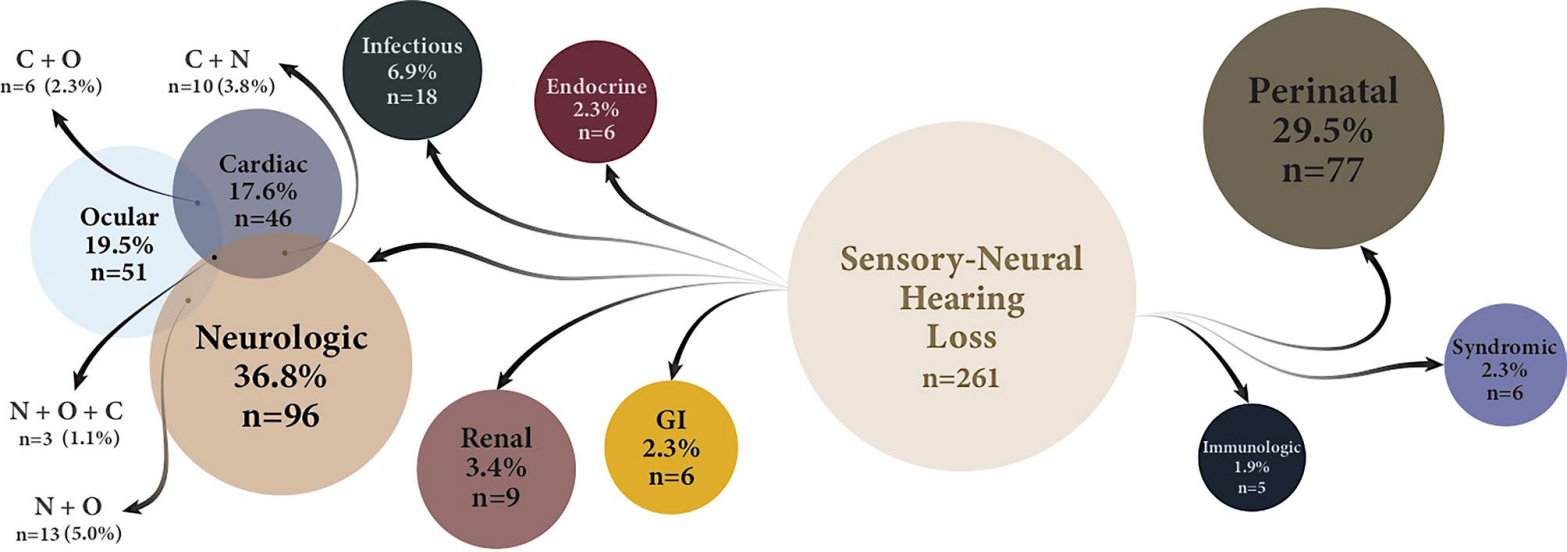
Distribution of systemic and ocular complications among pediatric candidates for CI due to bilateral SNHL (*N* = 261). Percentages reflect the proportion of the cohort affected by each category, based on retrospective chart review. The figure also illustrates the proportion of patients with co-occurring neurologic, cardiac and ocular findings

Jaundice was the most common prenatal complication observed in patients (*n* = 66, 25.3%). The most frequent neurological complications were facial nerve palsy (*n* = 51, 19.5%), seizure (*n* = 18, 6.9%), and motor delay (*n* = 11, 4.2%). Other observed complications were hypotonia, hydrocephalus, microcephaly, attention deficit hyperactivity disorder, and meningocele. Among cardiac abnormalities, valvular lesions were most frequent (*n* = 17, 6.5%), followed by patent foramen oval (*n* = 7, 2.7%), atrial septal defects (*n* = 5, 1.9%), patent ductus arteriosus (*n* = 5, 1.9%), and ventricular septal defect (*n* = 3, 1.1%). Electrical/conduction abnormalities (primarily prolonged QTc) occurred in 7 participants (2.7%). Dextrocardia and cardiomyopathy were each observed in 1 child (0.4% each). The prevalence of ophthalmologic findings was 19.5% (*n* = 51), of which 8.4% (*n* = 22) were refractive errors, and the rest were non-refractive errors. Strabismus and alignment disorders were the most common non-refractive errors (*n* = 10, 3.8%). Other ocular abnormalities observed included optic nerve abnormalities (*n* = 4, 1.5%), retinal dystrophy (*n* = 5, 1.9%), albinism or albinoid fundus changes (*n* = 5, 1.9%), Usher syndrome-related retinal changes (*n* = 4, 1.5%), and ectropion of the right lower eyelid (*n* = 1, 0.4%). Figure 1 illustrates the relative burden of systemic and ocular complications across the population, and highlights neurologic (36.8%) and perinatal (29.5%) complications as the most common comorbid findings, with ocular abnormalities present in nearly one in five patients (19.5%).

### Co-occurrence of ocular, cardiologic, and neurologic manifestations

To evaluate whether ocular findings co-occurred with cardiologic and neurologic manifestations, we examined the distribution of overlap among these three systems across all 261 patients (Table 2). Manifestation other than the three systems was present in 103 patients (39.5%). Isolated single-system involvement was the most common pattern; neurologic findings alone were observed in 70 patients (26.8%), ocular findings alone in 29 (11.1%), and cardiologic findings alone in 27 (10.3%). Two-system overlap was less frequent, occurring in 6 patients (2.3%) for ocular–cardiologic, 13 (5.0%) for ocular–neurologic, and 10 (3.8%) for cardiologic–neurologic findings. All three manifestations co-occurred in 3 patients (1.1%). (Table 2)

Pairwise chi-square tests showed no statistically significant association between any two systems: ocular and cardiologic findings (χ²(1) = 0.00, *p* = 0.996), ocular and neurologic findings (χ²(1) = 0.80, *p* = 0.372), and cardiologic and neurologic findings (χ²(1) = 1.74, *p* = 0.187).

To further evaluate this relationship, a multivariable binary logistic regression was performed with ocular findings as the outcome and cardiologic and neurologic involvement as predictors (*n* = 261). Neither cardiologic involvement (OR = 0.97, 95% CI 0.43–2.18, *p* = 0.946) nor neurologic involvement (OR = 0.74, 95% CI 0.38–1.43, *p* = 0.371) was significantly associated with the presence of ocular findings, consistent with the co-occurrence analysis above.

**Table 2:** Distribution of ocular, cardiologic, and neurologic manifestations

Category	*N*	% of 261
O only	29	11.1%
C only	27	10.3%
N only	70	26.8%
O + C	6	2.3%
O + N	13	5.0%
C + N	10	3.8%
O + C + N	3	1.1%
None of three	103	39.5%
**Total**	**261**	**100%**

O: ocular findings, C: cardiac findings, N: neurological findings

## Discussion

This study aims to determine the prevalence of ophthalmic findings in children with bilateral SNHL and an ABR threshold of ≥ 80 dB. All patients were candidates for CI. Identifying SNHL at a younger age allows for prompt interventions, reducing the developmental delay. However, this population faces additional complications. Neurological and ocular comorbidities can significantly impact a patient’s development. For this reason, it is advisable to screen them for a thorough neurologic and ophthalmologic examination. Refractive errors, strabismus, retinal, and optic nerve abnormalities are the most reported ocular comorbidities in SNHL patients [11, 12]. In a study by Sharma et al. [12], no difference was noted between the subcategorization of ocular findings and the severity of HL or laterality of SNHL. Leguire et al. found a higher prevalence of abnormalities in patients with severe HL [13]; however, this observation may be influenced by the higher prevalence of rubella infection in these patients.

Our observations revealed a prevalence of 2.4% for genetic syndromes. We also found a high prevalence of parental consanguinity in families of children with bilateral SNHL. A positive family history can also increase the risk of SNHL. Genetic counselling and, if necessary, genetic testing can determine the risk of such conditions in high-risk populations. Although the genetic causes of SNHL may seem rare, they are associated with a higher prevalence of ophthalmic findings compared to non-genetic SNHL [14]. It is also essential to consider that ocular abnormalities in this population may present years after the onset of SNHL. For example, the nyctalopia in Usher syndrome can happen years after the SNHL [15]. For these reasons, despite their rarity, the identification of such cases is of high importance, and clinicians must maintain a high level of suspicion.

Renal abnormalities were observed in 9 patients (3.4%) in this study. The eye and kidney share notable structural, developmental, and genetic pathways, suggesting a close biological link between ocular and renal disease [16]. Moreover, inner ear and kidney development are similarly governed by overlapping signaling and gene-expression programs, and both organs depend on comparable ciliary function, fluid-transport mechanisms, and a shared type IV collagen basement membrane network [17, 18]. Mutations affecting these common pathways have been implicated in syndromic associations such as branchio-oto-renal spectrum disorders and Alport syndrome, in which SNHL co-occurs with ocular anomalies and progressive renal disease [19, 20]. These developmental and physiological parallels offer a plausible biological basis for the co-occurrence of auditory, ocular, and renal abnormalities in SNHL and may warrant renal screening consideration in future prospective studies.

The co-occurrence analysis did not demonstrate a statistically significant clustering of ocular, cardiologic, and neurologic manifestations within this study. Both the pairwise chi-square comparisons and the multivariable logistic regression indicated that the presence of an abnormality in one organ system did not predict the presence of an abnormality in another, despite the embryological and etiological overlap proposed between these systems in syndromic and non-syndromic SNHL. This finding may reflect the heterogeneous and largely non-syndromic etiological basis of SNHL in our study, in which multisystem involvement may arise from distinct, independently acting causes rather than a shared underlying mechanism. Alternatively, the modest number of patients with overlapping manifestations (*n* = 3–13 per two- or three-system combination) may have limited statistical power to detect a true but modest association.

This independence has a direct and practical implication for clinical screening protocols in this population. Because ocular, cardiologic, and neurologic findings occurred independently of one another, the presence of an abnormality in one system cannot be used to infer the likelihood of abnormality in another, nor can it justify a more selective, risk-stratified approach to screening. These findings nonetheless underscore that ocular, cardiologic, and neurologic screening should be pursued independently and comprehensively in this population, rather than selectively guided by the presence of abnormality in another system.

Our study employed a cross-sectional design and included subjects from a single tertiary center. For this reason, we weren`t able to determine the prevalence of comorbidities in patients with mild or moderate SNHL. We were also unable to report the prevalence of each type of refractive error. Lack of universal genetic testing is another critical limitation that affects the prevalence of genetic syndromes in our study. Additionally, as this study evaluated ocular, cardiologic, and neurologic findings at the time of CI candidacy assessment, data on post-implantation rehabilitation status and language acquisition outcomes were not available, precluding assessment of whether visual cues or ocular abnormalities influence rehabilitation success in this population.

Despite these limitations, our findings highlight the importance of comprehensive multidisciplinary evaluation in children with SNHL, particularly those considered candidates for CI. Cardiovascular screening before surgery is a crucial consideration. Furthermore, neurologic and ocular examinations must be conducted to reduce the risk of developmental delay. Hypothyroidism is also another vital condition that may affect the typical developmental milestones of the SNHL population. Although we cannot determine whether renal, gastrointestinal, and immune complications are more prevalent in SNHL than in the general population, patients may experience them simultaneously. Overall management of a child with SNHL needs a multidisciplinary approach, and clinicians must have a high suspicion of other organ pathologies.

Future prospective, multicenter studies incorporating comprehensive genetic evaluation, long-term post-implantation rehabilitation, and language-acquisition outcomes are warranted. Such studies should determine whether the presence and timely correction of ocular abnormalities meaningfully influence visual-cue utilization, speech and language development, and overall rehabilitation success in children undergoing cochlear implantation.

## Conclusion

Ophthalmologic abnormalities were identified in a substantial proportion of pediatric candidates for CI with bilateral SNHL, with refractive errors, strabismus, retinal, and optic nerve abnormalities being the most frequent findings. These results support routine, comprehensive ophthalmic evaluation in this population, particularly before CI, to facilitate early detection of vision-threatening comorbidities, identify syndromic disease, and optimize developmental outcomes. Moreover, given that ocular, cardiologic, and neurologic manifestations occurred independently of one another in the exploratory analysis, screening for each system should be pursued comprehensively and in parallel rather than selectively triggered by findings in another system underscoring the need for a multidisciplinary evaluation protocol in pediatric candidates for CI.

## Data Availability

All available data are presented in this article and if more explanations are needed, contact the corresponding authors.

## Abbreviations

ABR: Auditory brainstem response
CI: Cochlear implantation
CI: Confidence interval
dB HL: Decibel hearing level
HL: Hearing loss
OR: Odds ratio
QTc: Corrected QT interval
SD: Standard deviation
SNHL: Sensorineural hearing loss
